# FetSAM: Advanced Segmentation Techniques for Fetal Head Biometrics in Ultrasound Imagery

**DOI:** 10.1109/OJEMB.2024.3382487

**Published:** 2024-03-27

**Authors:** Mahmood Alzubaidi, Uzair Shah, Marco Agus, Mowafa Househ

**Affiliations:** College of Science and EngineeringHamad Bin Khalifa University 370593 Doha 34110 Qatar

**Keywords:** Fetal Ultrasound Imaging, Image Segmentation, Prompt-based Learning, Prenatal Diagnostics, Ultrasound Biometrics

## Abstract

*Goal:* FetSAM represents a cutting-edge deep learning model aimed at revolutionizing fetal head ultrasound segmentation, thereby elevating prenatal diagnostic precision. *Methods:* Utilizing a comprehensive dataset–the largest to date for fetal head metrics–FetSAM incorporates prompt-based learning. It distinguishes itself with a dual loss mechanism, combining Weighted DiceLoss and Weighted Lovasz Loss, optimized through AdamW and underscored by class weight adjustments for better segmentation balance. Performance benchmarks against prominent models such as U-Net, DeepLabV3, and Segformer highlight its efficacy. *Results:* FetSAM delivers unparalleled segmentation accuracy, demonstrated by a DSC of 0.90117, HD of 1.86484, and ASD of 0.46645. *Conclusion:* FetSAM sets a new benchmark in AI-enhanced prenatal ultrasound analysis, providing a robust, precise tool for clinical applications and pushing the envelope of prenatal care with its groundbreaking dataset and segmentation capabilities.

## I. Introduction

In The transformative realm of biomedical imaging, fetal ultrasound serves as a critical juncture between technology and healthcare [1]. It has revolutionized prenatal care by providing a non-invasive window into the womb, enabling real-time monitoring of fetal growth and development [2]. The significance of standardizing quantification and achieving precise fetal head segmentation goes beyond technicality; it serves as a beacon for advancing prenatal care [3]. Reducing variability in data collection is crucial for reliable monitoring across multiple scans. Furthermore, the cost-effectiveness and widespread accessibility of ultrasound imaging make it a powerful tool for global healthcare improvement [4]. Future integration of AI-enabled tissue segmentation with ultrasound imaging could catalyze earlier interventions, safer deliveries, and healthier beginnings, positioning this research as an essential cornerstone in prenatal care [5].

Accurate estimation of fetal age and weight is pivotal in monitoring standard prenatal development [1]. Ultrasound imaging facilitates the non-invasive observation of the fetus, encompassing the brain structures. Modern advancements in image processing and segmentation methods have paved the way for the automatic delineation and measurement of essential fetal brain regions from ultrasound images [6]. In particular, segmenting the fetal brain, cavum septum pellucidum (CSP), and lateral ventricles (LV) from ultrasound images can offer indispensable biomarkers for gestational age and fetal growth models [1]. The CSP is a crucial midline fluid-filled cavity, the size of which fluctuates significantly during gestation [7], while the size of the LV augments with progressing gestational age [8]. Thus, a quantitative analysis of the CSP and LV dimensions derived from segmented ultrasound images can offer profound insights into the developmental milestones of the fetus. Moreover, the total volume of the brain tissue, determined from the automatic segmentation of the brain, has exhibited a strong correlation with the fetal weight [9]. By amalgamating the measures from the segmented multiple brain structures, sturdy machine learning models can be devised to determine fetal age and weight from ultrasound images with automation and efficiency [10]. The automation of this analytical procedure has the potential to enhance prenatal risk assessments and ameliorate outcomes by promoting more comprehensive and quantitative surveillance of fetal development [11].

**Fig. 1.**
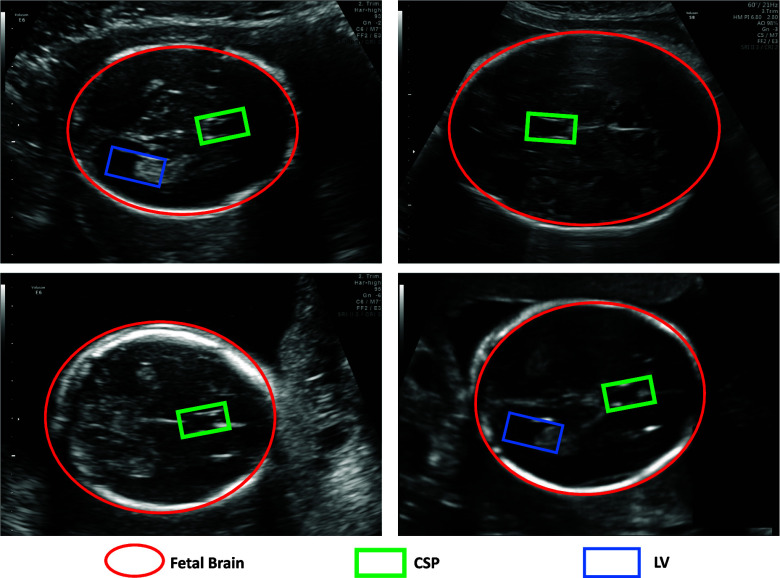
Illustration depicting the location of Fetal Brain, CSP, and LV in the ultrasound image.

The precise segmentation of fetal head ultrasound images into four paramount categories–background, fetal brain, CSP, and LV–is at the heart of this study. Each category plays a pivotal role in deriving essential biometrics. Automated tools have made it possible to segment vital anatomical structures, such as the fetal skull and brain, paving the way for the accurate and efficient determination of crucial biometric measures, including the head circumference [12], [13]. Refer to Fig. 1 for an illustration that depicts the locations of the Fetal Brain, CSP, and LV in the ultrasound image.

The background class, essentially all ultrasound data outside the fetal skull, allows for the automatic cropping of images to focus on the region of interest, namely the fetal head [14]. The fetal brain class encapsulates all brain tissue within the skull. Accurate delineation of this class enables the measurement of head circumference, a critical indicator of fetal growth and developmental milestones [6]. Further, segmenting internal structures like the CSP and LV provides deeper insights into fetal neurodevelopment [3]. The CSP, a naturally occurring division between the left and right hemispheres, can serve as an early indicator of potential brain abnormalities [15]. The LV, on the other hand, are fluid-filled cavities related to conditions such as ventriculomegaly and neural tube defects [16]. Accurate segmentation of these structures is critical for monitoring fetal brain development, thereby enabling better-informed parental decisions and preparing the clinical team for post-birth interventions.

One of the most salient challenges in fetal ultrasound imaging is the quality of the images themselves [17]. Ultrasound images often suffer from speckle noise, low contrast, and ambiguous boundaries, making it difficult to segment anatomical structures like the brain, CSP, and LV [12], [18]. Wu et al. [19] highlight the importance of CSP, an essential structure for normal fetal brain development, and discuss the challenges of manual measurement. They propose a data-driven system using a novel network called CA-Unet to segment CSP and other related structures, achieving high precision and Dice scores. Similarly, Coronado-Gutiérrez et al. [20] introduce a pipeline to automatically delineate and measure fetal brain structures, offering a comprehensive solution for assessing fetal brain development and anomalies. Alzubaidi et al. [21] introduced the ETLM framework, which leverages ensemble learning for fetal brain segmentation and employs regression models to estimate fetal age and weight based on biometric measurements.

The CSP and LV are particularly challenging due to their small sizes and low visibility, often occupying only a few pixels in 2D ultrasound slices [13]. The fetal brain undergoes rapid development, complicating the segmentation task further due to fluctuations in visibility at different gestational stages [18], [22].

Classical models like active contours and atlas-based approaches have been widely used but often struggle with the aforementioned challenges. For instance, active contours often fail to converge to the correct boundaries [18]. The advent of deep learning has brought a revolution in this domain. U-Net architectures, for example, have shown promise but are also subject to limitations like overfitting and lack of generalizability [18].

In addressing the challenges of fetal brain segmentation, we explore a spectrum of methodologies, significantly benefiting from advancements in 2D fetal ultrasound neuroimaging. While, recent studies have demonstrated notable success in enhancing segmentation accuracy and reliability.

Zeng et al. [23] introduce a deep learning method specifically tailored for fetal ultrasound image segmentation, achieving remarkable precision in head circumference biometry. Their approach, incorporating attention-gated modules into a V-Net model, underscores the effectiveness of deep supervision and attention mechanisms in focusing on relevant features, thus improving segmentation accuracy.

Similarly, Zhao et al. [24] present TransFSM, a hybrid deep learning framework for automated segmentation and biometric measurement, which employs a convolutional neural network encoder alongside a global transformer module. This innovative combination facilitates the learning of long-range dependencies, enhancing the model's ability to segment multiple fetal anatomies accurately from 2D ultrasound images.

In addition, Wu et al. [19] detail a novel approach for the automatic segmentation and measurement of the CSP using a U-Net-based network augmented with a channel attention module. Their results indicate superior performance in CSP segmentation, contributing valuable insights into fetal neural development assessment.

Drawing on the latest advancements in 2D fetal ultrasound imaging, our study methodically integrates advanced segmentation techniques to address critical challenges within the field. Through the strategic implementation of class weighting, bespoke loss functions, and comprehensive data augmentation, we precisely target dataset imbalances, with a particular focus on the LV class. This multifaceted approach not only capitalizes on foundational research to enhance segmentation accuracy and expand its generalizability but also highlights areas ripe for further exploration, as evidenced by our targeted ablation studies. Our commitment to refining fetal ultrasound segmentation is clear, marking a significant step forward in prenatal diagnostic technologies.

In the realm of fetal brain segmentation, the scarcity of annotated data poses a significant challenge, prompting a shift towards innovative approaches such as weakly supervised and semi-supervised methods. These methods, exemplified by the work of Zheng et al. [25] with their ‘shadow confidence maps' and Yang et al. [26]'s use of attention mechanisms and RNNs, represent a broader endeavor to refine segmentation accuracy under data constraints. Similarly, hybrid models like those developed by Chen et al. [27] and Yang et al. [28] blend traditional segmentation techniques with deep learning advancements to overcome these limitations.

Despite these efforts, as evidenced by the achievements in Dice scores for CSP and LV segmentation reported by Huang et al. [18], the quest for highly accurate, robust, and generalizable segmentation methods remains ongoing. This backdrop of continuous exploration and innovation in addressing data scarcity and enhancing segmentation precision frames the context for our study. Our work contributes to this dynamic field by introducing FetSAM, a model designed to advance the state-of-the-art in fetal head ultrasound image segmentation. By situating our contributions within this evolving landscape, we aim to underscore the significance of our advancements and the potential for further research to navigate the complexities of fetal brain, CSP, and LV segmentation.

In addressing the significant challenges of fetal brain, CSP, and LV segmentation, such as dataset scarcity, image noise, and the minute size of anatomical structures, our methodology is deliberately designed for precision. By constructing a custom dataset that includes detailed structures of CSP and LV, we tackle the issue of data scarcity head-on. To mitigate inherent noise and enhance segmentation precision, we've implemented an extensive data augmentation strategy, applying ten different techniques that simulate real-world imaging challenges, thus preparing our model for a wide range of imaging conditions. Recognizing the challenges posed by the small size and class imbalance of CSP and LV, we developed a sophisticated combined loss function that integrates weighted DiceLoss and Weighted Lovasz loss mechanisms. This approach ensures that these critical, yet smaller, structures receive adequate attention during the learning process, significantly boosting segmentation accuracy. Additionally, our use of prompt-based segmentation acts as a magnifying glass, bringing regions of interest into clearer focus, which is crucial for the detailed segmentation of CSP and LV, thereby enhancing the accuracy of biometric measurements. Our comprehensive methodology has been rigorously validated against ten state-of-the-art segmentation models, including those based on modern mixed transformer architectures, ensuring our approach's transparency and superiority in addressing these unique challenges. Guided by these challenges, we've meticulously developed a comprehensive approach, outlined below, to not only address these specific issues head-on but also to set new benchmarks in the accuracy and reliability of fetal brain, CSP, and LV segmentation.

1) New Fetal Head Ultrasound Dataset: We address data scarcity by creating a unique dataset with comprehensive coverage of CSP and LV structures across various brain planes, ensuring robust training and validation.
2) Data Augmentation: To combat image noise and ambiguities, we implemented advanced data augmentation, applying techniques that closely mimic real-world imaging variations, thereby enhancing model adaptability and generalization capabilities.
3) Class Weight Calculations and Loss Mechanism: Acknowledging the small size and imbalance of CSP and LV, we introduce a custom combined loss function, integrating weighted DiceLoss and Weighted Lovasz loss, to prioritize precision in segmenting these critical but underrepresented structures.
4) FetSAM (Fetal Segment Anything Model): This novel prompt-based model excels in discerning anatomical structures with unmatched accuracy, evidenced by superior metrics like a Dice Similarity Coefficient (DSC) of 0.90117, Hausdorff Distance (HD) of 1.86484, and Average Surface Distance (ASD) of 0.46645, challenging conventional segmentation approaches.
5) Comparative Analysis: Our extensive evaluation against ten leading models, including U-net, DeepLabV3, and Segformer, highlights FetSAM's advanced performance, marking a significant leap in segmentation technology for prenatal care.

## II. Materials and Methods

**Fig. 2.**
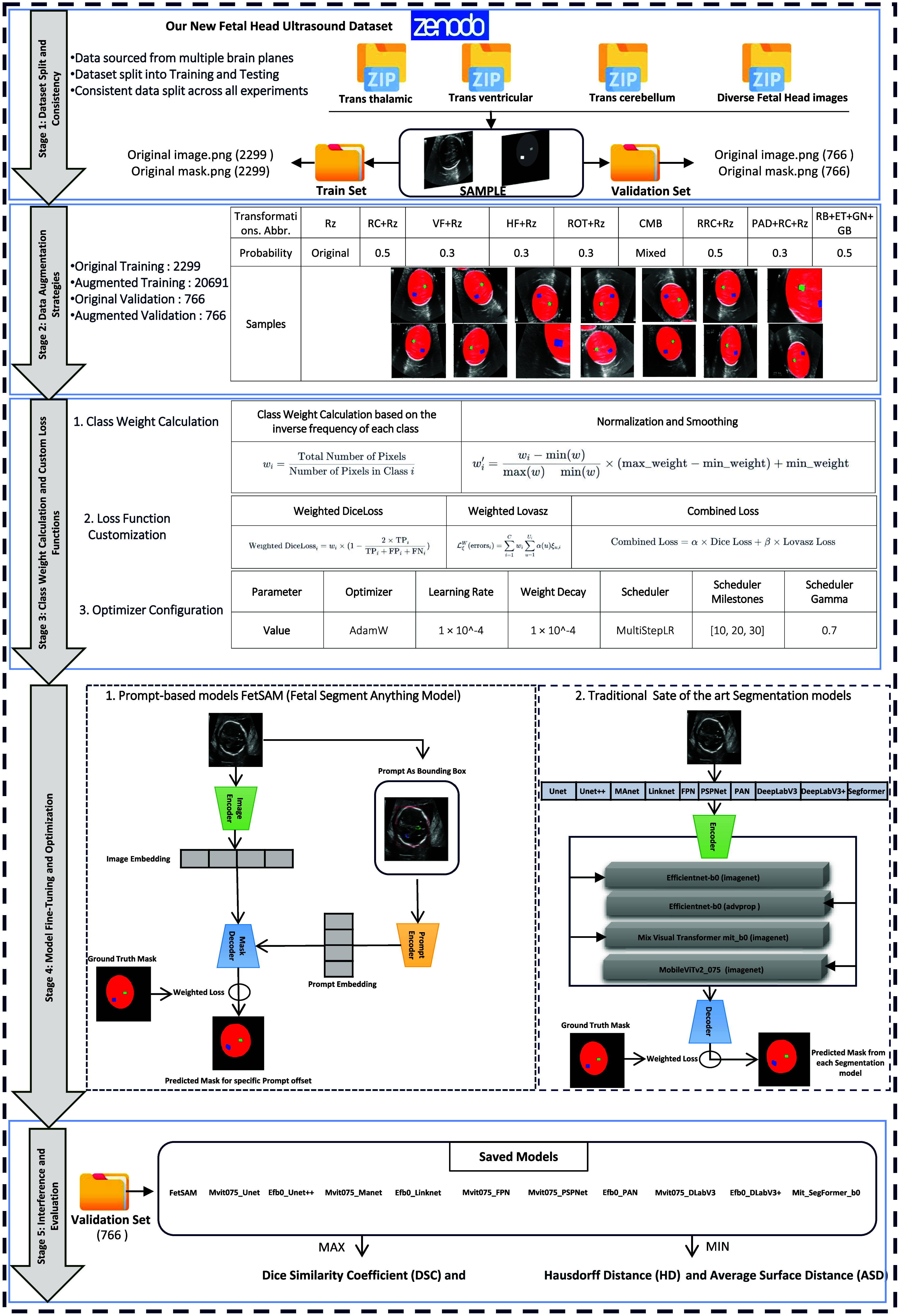
Schematic of the optimized pipeline for comparing FetSAM with state-of-the-art models in multi-class segmentation of fetal head ultrasound imaging.

Fig. 2 illustrates our optimized pipeline for comparing FetSAM with state-of-the-art models in multi-class segmentation of fetal head ultrasound imaging. The pipeline encompasses five critical stages: Dataset Split and Consistency, Data Augmentation Strategies, Class Weight Calculation and Custom Loss, Model Fine-Tuning and Optimization Functions, and Inference and Evaluation. Starting with our unique fetal head ultrasound dataset, the pipeline integrates advanced techniques such as extensive data augmentation, class weight calculation based on inverse frequency, custom loss mechanisms, and fine-tuning optimization techniques. The pipeline culminates in a comparative analysis of FetSAM against established segmentation models like U-Net, DeepLabV3, and Segformer, assessed using a variety of metrics including DSC and HD. The following subsections will delve into each stage of this pipeline, from dataset description and augmentation strategies to model architecture and evaluation metrics.

### A. Dataset Description and Split

Our research utilizes a comprehensive dataset compiled from two public repositories, featuring four distinct ultrasound fetal head planes: Trans thalamic, Trans ventricular, Trans cerebellum, and Diverse Fetal Head images. Comprising 3,832 high-resolution images, this dataset emphasizes critical fetal brain structures, including the CSP and LV. To facilitate broad computational tool compatibility, data is available in 11 widely accepted formats, verified by a rigorous dual-stage process involving multiple domain experts.

For experimental consistency, a custom script was used to segment the dataset across different ultrasound planes, allocating 60% (2,299 images) for training. The remaining 40% was divided equally between a validation set (20%, 766 images) and an untouched test set (20%, 767 images), reserved for future clinical validation. This test set remains unused in the current study to ensure the model's future clinical applicability and effectiveness are assessed independently. The validation set includes a balanced class distribution of background, brain, CSP, and LV instances, offering a solid basis for model evaluation. This distribution and strategic data split underscore our commitment to a methodical and future-oriented approach to research validation. For further details on this dataset, readers are referred to the original publication by Alzubaidi et al. [29].

### B. Data Augmentation Strategies

To bolster the robustness of our model and effectively utilize our training dataset, we employed a comprehensive data augmentation strategy. While the original training dataset consisted of 2,299 images, these augmentation techniques expanded it to a total of 20,691 images. Importantly, the validation dataset was left unaugmented to ensure an unbiased evaluation of the model's performance.

Our data augmentation pipeline comprised a series of transformations, each designed to simulate real-world variations that the model might encounter. Below are the transformations applied:

1) Original Image Resize (Rz): The images were resized to 256 × 256 pixels. No other transformations were applied to maintain the originality of the dataset.
2) Random Crop (RC+Rz): Random crops of 128 × 128 pixels were taken from the resized images, which were then resized back to 256 × 256 pixels.
3) Vertical Flip (VF+Rz): A vertical flip was applied with a probability of 0.3.
4) Horizontal Flip (HF+Rz): A horizontal flip was applied with a probability of 0.3.
5) Rotation (ROT+Rz): The images were rotated within a limit of 30 degrees, applied with a probability of 0.3.
6) Combined Transformations (CMB): A chain of transformations, including random crop, vertical flip, horizontal flip, and rotation, was applied.
7) Random Resized Crop (RRC+Rz): The images were randomly resized within a scale of 1.2 to 1.4, and then cropped to 256 × 256 pixels.
8) Padding and Random Crop (PAD+RC+Rz): Padding was applied to increase the image size, followed by a random crop to bring it back to 256 × 256 pixels.
9) Advanced Transformations (RB+ET+GN+GB): This set of sophisticated techniques was designed to simulate complex variations and artifacts commonly found in ultrasound images. All transformations were applied with a probability of 0.5 and include:

• *Brightness and Contrast Adjustments*(RB)*:* Random adjustments simulate variations in lighting and visibility.
• *Elastic Transformations*(ET)*:* Elastic deformations with an alpha parameter of 1 and a sigma parameter of 50 mimic natural variations in tissue elasticity.
• *Gaussian Noise*(GN)*:* Noise with a variance limit ranging from 10.0 to 50.0 simulates the speckle noise commonly found in ultrasound images.
• *Gaussian Blur*(GB)*:* A blur with a limit ranging from 3 to 7 simulates the effects of blurring due to factors like patient movement or low-quality imaging devices.

These advanced transformations aim to train the model to be more resilient to real-world variations, thereby enhancing its generalizability and robustness. Implemented using the Albumentations library for consistency.

### C. Class Weight Calculation and Custom Loss Mechanism

#### 1) Class Weight Calculation

To address the challenges posed by our dataset, we opted for a specific type of class weighting for multiple reasons. First, our dataset is imbalanced; the number of instances for the “background” and “fetal brain” classes significantly outnumber those for the “CSP” and “LV” classes. Second, the geometric characteristics of the classes also vary; while classes like “CSP” and “LV” are represented by small rectangles, the “fetal brain” class is represented by a larger ellipse. This class-weighting approach aims to compensate for these disparities, enhancing the model's ability to accurately segment all classes.

1) *Initialize Class Counts:* We initiate an array class_counts with zeros to store the count of each class. The size of this array is the number of classes $n_{\text{classes}}$.
  \begin{equation*}
\text{class}\_{\text{counts}} = \mathbf {0} \in \mathbb {R}^{n_{\text{classes}}}
\end{equation*}
2) *Iterate Over the Dataset:* For each batch of masks in the data loader, we convert the one-hot encoded masks to class labels.
  \begin{equation*}
\text{masks} = \arg \max (\text{masks}\_{\text{one}}\_{\text{hot}}, \text{dim=1})
\end{equation*}
3) *Update Class Counts:* We then count the occurrences of each class in these masks and update class_counts.
  \begin{equation*}
\text{unique, counts} = \text{np.unique}(\text{masks}, \text{return}\_{\text{counts=True}})
\end{equation*}
  \begin{equation*}
\text{class}\_{\text{counts}}[\text{unique}] += \text{counts}
\end{equation*}
4) *Handle Zero Counts:* To avoid division by zero, we replace any zero count with one.
  \begin{equation*}
\text{class}\_{\text{counts[class}}\_{\text{counts = 0]}} = 1
\end{equation*}
5) *Calculate Raw Weights:* The raw class weights are calculated as the inverse frequency of each class normalized by the sum of these inverse frequencies.
  \begin{align*}
\text{total}\_{\text{count}} =& \sum _{i=1}^{n_{\text{classes}}} \text{class}\_{\text{counts}}[i] \\
\text{class}\_{\text{weights}} =& \frac{\text{total}\_{\text{count}}}{\text{class}\_{\text{counts}}} \\
\text{class}\_{\text{weights}} =& \frac{\text{class}\_{\text{weights}}}{\sum \text{class}\_{\text{weights}}}
\end{align*}
6) *Smoothing and Normalization:* We smooth the weights to ensure they fall within a specified range $[ \text{min}\_{\text{weight}}, \text{max}\_{\text{weight}} ]$.
  \begin{align*}
\!\!\text{weights} =& \Bigg(\!\! \frac{\text{smoothed}\_{\text{weights}} {-} \min (\!\text{smoothed}\_{\text{weights}}\!)}{\max (\!\text{smoothed}\_{\text{weights}}\!)} \\
& - \min (\text{smoothed}\_{\text{weights}}) \Bigg) \\
& \times (\text{max}\_{\text{weight}}\! - \!\text{min}\_{\text{weight}})\! +\! \text{min}\_{\text{weight}} \tag{1}
\end{align*}
7) *Return Weights:* Finally, we return the normalized class weights as a list and optionally as a dictionary mapped to class names.

#### 2) Custom Loss Mechanism

To address the issue of class imbalance and the distinct geometric characteristics of various classes in our dataset, we have employed the class weights derived from the data analysis phase to construct a bespoke loss function. This function intensifies the model's focus on the smaller and less prevalent classes like CSP and LV. Specifically, with class weights like [0.1,0.1,0.9,0.7], our custom loss function will allocate more attention to classes with larger weights, ensuring that difficult-to-segment classes are prioritized during training. Selecting the most effective loss functions was crucial for the optimization of our model. We assessed several loss functions such as JaccardLoss, DiceLoss, TverskyLoss, FocalLoss, and LovaszLoss, alongside their possible combinations. Through thorough manual hyperparameter tuning and empirical evaluation, we identified that an amalgamation of Weighted Dice Loss and Weighted Lovasz Loss yielded the most promising segmentation results.

The Weighted Dice Loss $L_{\text{Dice}}$ is formulated as follows:

\begin{equation*}
L_{\text{Dice}} = 1 - \frac{2 \times \sum _{i=1}^{N} (y_{i} \times p_{i} \times w_{i})}{\sum _{i=1}^{N} (y_{i} + p_{i}) \times w_{i}} \tag{2}
\end{equation*}

where $y_{i}$ and $p_{i}$ denote the ground truth and the predicted probabilities for pixel $i$, $w_{i}$ represents the class weight for pixel $i$, and $N$ is the total number of pixels.

Lovasz loss is an extension of subgradient methods for convex optimization to the problem of optimizing the mean intersection-over-union (IoU) measure, which is nonlinear and non-differentiable. In the context of binary segmentation, the Lovasz hinge loss effectively leverages the convex hull of the Jaccard index, optimizing the IoU metric directly [30].

The Weighted Lovasz Loss $L_{\text{Lovasz}}$ can be depicted as:

\begin{equation*}
L_{\text{Lovasz}} = \sum _{i=1}^{N} \text{Lovasz}(y_{i}, p_{i}) \times w_{i} \tag{3}
\end{equation*}

where $\text{Lovasz}(y_{i}, p_{i})$ is the Lovasz hinge loss for the true label $y_{i}$ and the predicted label $p_{i}$, and $w_{i}$ is the class weight for pixel $i$.

The Combined Loss $L_{\text{Combined}}$, a synthesis of the two losses, is calculated as:

\begin{equation*}
L_{\text{Combined}} = \alpha \times L_{\text{Dice}} + \beta \times L_{\text{Lovasz}} \tag{4}
\end{equation*}

where $\alpha$ and $\beta$ are the hyperparameters that modulate the influence of each loss term on the final combined loss. In our fine-tuned model, we have set both $\alpha$ and $\beta$ to 0.5, reflecting the equal contribution of each loss term to the final loss value.

By merging these losses, our goal was to exploit the distinctive advantages of both Dice and Lovasz losses, while considering class weights, to achieve a segmentation model that is both accurate and sensitive to the nuances of each class.

#### 3) Optimizer Configuration

During the developmental phase of our model, various optimizers and learning rate schedulers, including the standard Adam optimizer, were extensively explored and tested. Our empirical evaluations, coupled with a rigorous process of trial and error, helped in steering the choice towards the most optimal configuration for our specific application.

In this study, we employ the AdamW optimizer, which has manifested superior performance in terms of model convergence and generalization. The learning rate and weight decay are both set at $1 \times 10^{-4}$. To further refine the optimization process, we utilize a MultiStepLR scheduler that dynamically adjusts the learning rate at predefined epochs $[10, 20, 30]$ with a gamma value of 0.7. This intricate setup ensures a harmonious blend of swift convergence and robust model generalization, vital for the intricate task at hand.

### D. Introduction to Fetal Segment Anything Model (FetSAM)

In our study, we leverage the Segment Anything Model (SAM) [31], a promptable foundation model developed for generic image segmentation tasks. SAM consists of three main components: an Image Encoder, a Prompt Encoder, and a Mask Decoder (see Fig. 2). The Image Encoder is responsible for processing the input image and generating a set of image features. Simultaneously, the Prompt Encoder produces a prompt embedding based on the input prompt, usually a spatial or textual clue. The Mask Decoder then utilizes both the image features and prompt embedding to generate a segmentation mask.

To adapt SAM for fetal head ultrasound imaging, we introduce FetSAM (Fetal Segment Anything Model). We fine-tune the mask decoder component of SAM using our custom loss function, which incorporates class weights to address the imbalanced nature of our dataset. We also employ an extensive data augmentation strategy to increase the diversity and size of our training dataset.

The architecture of FetSAM is similar to SAM but includes a few key customizations. The Image Encoder and Prompt Encoder produce embeddings that are fed into the Mask Decoder. Unlike in SAM, our Mask Decoder is trained to be more sensitive to the fetal brain structures of interest, particularly Fetal brain, CSP and LV, by incorporating our custom loss mechanism. This enables FetSAM to generate more accurate and clinically relevant segmentation masks for prenatal care (Fig. 2).

To generate prompt bounding boxes from the labeled masks, we designed an algorithm that iterates through each class channel in a one-hot encoded mask to compute the minimum and maximum coordinates that define the bounding box for each class.

Algorithm 1: **Algorithm for Deriving Prompt Bounding Boxes.**
**Require:**One-hot encoded mask $\text{one}\_{\text{hot}}\_{\text{mask}}$ of shape $C \times H \times W$
**Require:**Number of classes $\text{num}\_{\text{classes}}$
**Require:**Threshold $\text{threshold}$
**Require:**Offset $\text{offset}$
**Ensure:**Dictionary $\text{bounding}\_{\text{boxes}}$ containing bounding boxes for each class
1: Initialize $\text{bounding}\_{\text{boxes}}$ as empty dictionary
2: **for** each class $i$ in $C$ **do**
3: $y_{\text {indices}}, x_{\text {indices}} \leftarrow$ find where $\text{one}\_{\text{hot}}\_{\text{mask}}[i] > \text{threshold}$
4: **if** $y_{\text {indices}}.size = 0$ OR $x_{\text{indices}}.size = 0$ **then**
5: Continue to the next iteration
6: **end if**
7:
$x_{\text{min}}, x_{\text{max}} \leftarrow \min (x_{\text {indices}}), \max (x_{\text {indices}})$

8:
$y_{\text{min}}, y_{\text{max}} \leftarrow \min (y_{\text {indices}}), \max (y_{\text {indices}})$

9: Apply offset to $x_{\text{min}}, x_{\text{max}}, y_{\text{min}}, y_{\text{max}}$
10:
$\text{bbox} \leftarrow [x_{\text{min}}, y_{\text{min}}, x_{\text{max}}, y_{\text{max}}]$

11:
$\text{bounding}\_{\text{boxes}}[i] \leftarrow \text{bbox}$

12: **end for**
13: **for** each class $i$ in $\text{num}\_{\text{classes}}$ **do**
14: **if** $i$ not in $\text{bounding}\_{\text{boxes}}$ **then**
15:
$\text{bounding}\_{\text{boxes}}[i] \leftarrow [0, 0, 0, 0]$

16: **end if**
17: **end for**
18: **return** $\text{bounding}\_{\text{boxes}}$

This algorithm 1 deriving prompt bounding boxes that takes a one-hot encoded mask $\text{one}\_{\text{hot}}\_{\text{mask}}$ with dimensions $C \times H \times W$, where $C$ represents the number of classes, $H$ is the height, and $W$ is the width of the mask. Additional inputs to the algorithm include the number of classes $\text{num}\_{\text{classes}}$, a threshold value $\text{threshold}$, and an offset $\text{offset}$ to adjust the bounding boxes. The algorithm outputs a dictionary $\text{bounding}\_{\text{boxes}}$ containing bounding boxes for each class.

Initially, $\text{bounding}\_{\text{boxes}}$ is set as an empty dictionary. The algorithm then iterates over each class $i$ in $C$ to find the $y$ and $x$ indices where the mask value exceeds the threshold. If either set of indices is empty, the algorithm continues to the next iteration. Otherwise, it calculates the minimum and maximum $x$ and $y$ indices and applies the given offset to them. The resulting bounding box $\text{bbox} = [x_{\text{min}}, y_{\text{min}}, x_{\text{max}}, y_{\text{max}}]$ is stored in $\text{bounding}\_{\text{boxes}}$ under the corresponding class $i$.

After traversing all the classes in $C$, the algorithm checks for any classes missing in $\text{num}\_{\text{classes}}$. For any such missing classes, it assigns a default bounding box $[0, 0, 0, 0]$. Finally, the algorithm returns $\text{bounding}\_{\text{boxes}}$, thus providing a comprehensive set of bounding boxes for each class based on the input segmentation mask.

### E. Traditional Sate of the Art Segmentation Models

In this section, we delineate the procedure employed for the fine-tuning of various conventional segmentation models. Our approach involved a systematic evaluation of multiple encoder architectures for each segmentation model to ascertain the most efficacious combinations.

#### 1) Encoder Models

In this subsection, we present the encoder architectures that were evaluated for their efficacy in segmentation tasks see (Fig. 2. Four distinct encoder models were chosen based on their computational efficiency, performance, and architectural innovations. They are as follows:

1) *EfficientNet-B0 (ImageNet) [32]:* EfficientNet is a convolutional neural network architecture that scales up CNNs in a principled way using compound scaling. EfficientNet-B0 is a lightweight variant that achieves strong performance on ImageNet while being very computationally efficient.
2) *EfficientNet-B0 (AdvProp) [33]:* This model retains the EfficientNet-B0 architecture but incorporates the AdvProp training methodology. The latter dynamically adjusts the scaling factor for adversarial examples during training, thereby enhancing both robustness and accuracy.
3) *MiT-B0 (ImageNet) [34]:* The Mix Transformer (MiT) replaces the standard convolutional backbone with a transformer encoder, enabling the modeling of longer-range dependencies in images. The B0 variant maintains high ImageNet accuracy while being computationally efficient.
4) *MobileViTv2_075 (ImageNet) [35]:* Designed for mobile applications, MobileViTv2 employs an inverted bottleneck convolutional stem and depthwise convolutions in its transformer blocks. The _075 variant further improves efficiency with a width multiplier of 0.75x.

#### 2) Segmentation Model Selection and Optimization

In this subsection, we delve into the process of fine-tuning traditional segmentation models for our specific task as we illustrated early in Fig. 2. We experimented with ten different segmentation architectures, each paired with multiple encoder models. The goal was to identify the optimal configuration that achieves high segmentation performance while maintaining computational efficiency. Follow is a brief about Each Segmentation Model with Additional Information:

1) *U-Net (Mvit075_Unet) [36]:* The U-Net architecture consists of an encoder network followed by a decoder network with skip connections between them. The encoder extracts features and the decoder upsamples and reconstructs the segmentation mask. The skip connections retain fine-grained information. In our case, the model is paired with the MobileViTv2_075 encoder and has an encoder depth of 5.
2) *U-Net++ (Efb0_Unet++) [37]:* U-Net++ improves on U-Net by redesigning the decoder component with a series of nested, dense skip pathways to better leverage encoder features at multiple scales. This enhances segmentation detail. We optimized this model with the EfficientNet-B0 encoder.
3) *MA-Net (Mvit075_Manet) [38]:* MA-Net introduces a new attention refinement module between the encoder and decoder that models inter-dependencies between encoder features. This improves feature representation and segmentation accuracy. The model utilizes the MobileViTv2_075 encoder with an encoder depth of 5.
4) *LinkNet (Efb0_Linknet) [39]:* LinkNet is built from an encoder network followed by a decoder. The key aspect is linking the encoder and decoder directly with residual connections that improve information flow. Our implementation employs the EfficientNet-B0 encoder.
5) *FPN (Mvit075_FPN) [40]:* Feature Pyramid Network (FPN) creates a pyramid of hierarchical encoder features. Decoder stages then upsample and merge these multi-scale features to capture both local and global context. The model uses the MobileViTv2_075 encoder.
6) *PSPNet (Mvit075_PSPNet) [41]:* Pyramid Scene Parsing Network uses pyramid pooling modules after the encoder to aggregate different-region contextual information. This global context aids challenging segmentation tasks. It is paired with the MobileViTv2_075 encoder.
7) *PAN (Efb0_PAN) [42]:* Path Aggregation Network adds a bottom-up path augmentation through adaptive feature pooling to complement the original top-down feature propagation in the decoder. The model uses the EfficientNet-B0 encoder.
8) *DeepLabV3 (Mvit075_DLabV3) [43]:* DeepLabV3 employs atrous convolution in the encoder and decoder to extract dense feature maps. It also uses pyramid pooling to encode multi-scale context. The model uses the MobileViTv2_075 encoder.
9) *DeepLabV3+ (Efb0_DLabV3+) [44]:* DeepLabV3+ enhances DeepLabV3 by adding a decoder module to refine the segmentation results, especially along object boundaries. It's optimized with the employs the EfficientNet-B0 encoder with AdvProp pre-trained.
10) *SegFormer (Mit_SegFormer_b0) [34]:* SegFormer employs a hierarchical Transformer encoder, specifically MiT-B0, in combination with a lightweight decoder. This configuration achieves robust performance without the need for dense prediction layers.

### F. Models Evaluation

In this section, we elaborate on the evaluation metrics employed for assessing the segmentation performance of the proposed FetSAM model alongside other traditional segmentation algorithms. Each metric is calculated separately for the four segmented classes: background, Brain, CSP, and LV. Subsequently, the mean values are also calculated to furnish a comprehensive evaluation of the model's performance. All models are validated using the same dataset to ensure a fair comparison.

#### 1) Metrics Optimized for Maximum Values

• *Dice Similarity Coefficient (DSC):* The DSC is defined as
  \begin{equation*}
DSC(A, B) = \frac{2 \times |A \cap B|}{|A| + |B|}
\end{equation*}
  where $A$ and $B$ are the sets of pixels belonging to the predicted and ground truth masks, respectively. $|A \cap B|$ denotes the size of the intersection of the two sets. The DSC ranges from 0 to 1, where a higher value indicates better similarity.

#### 2) Metrics Optimized for Minimum Values

• *Hausdorff Distance (HD):* The HD is defined as the maximum of two directed Hausdorff distances:
  \begin{equation*}
HD(A, B) = \max \left(\sup _{a \in A} \inf _{b \in B} d(a, b), \sup _{b \in B} \inf _{a \in A} d(a, b) \right)
\end{equation*}
  where $\sup$ and $\inf$ denote the supremum and infimum, and $d(a, b)$ is the Euclidean distance between points $a$ and $b$. A lower HD indicates better similarity.
• *Average Surface Distance (ASD):* The ASD is the mean of all the surface distances between the border points of the predicted and ground truth masks. It is defined as
  \begin{equation*}
ASD(A, B) = \frac{1}{|A_{\text{border}}|} \sum _{a \in A_{\text{border}}} \min _{b \in B_{\text{border}}} d(a, b)
\end{equation*}
  where $A_{\text{border}}$ and $B_{\text{border}}$ are the sets of border points in $A$ and $B$, respectively. A lower ASD indicates a better match.

### G. Experimental Environment

The experiments for this study were conducted on a robust computational setup powered by a 12th Gen Intel(R) Core(TM) i7-12700KF processor, complemented by 128 GiB of system memory. The graphics-intensive tasks were handled by an NVIDIA GeForce RTX 3090 graphics card, equipped with 24 GB of GDDR6X VRAM.

In terms of software frameworks, we primarily relied on PyTorch for constructing the model architectures, training, and evaluation. To streamline the training process and for better experiment tracking, PyTorch Lightning was utilized. For leveraging pre-trained models and additional utilities, we also made use of the Hugging Face Transformers library.

To ensure optimal model performance and avoid overfitting, training was monitored with an early stopping mechanism, configured with a patience of 5 epochs. This approach allowed us to halt the training process when the model's performance ceased to improve on the validation set, thus saving computational resources and time.

## III. Results

In this section, we present a comprehensive evaluation of the proposed FetSAM model against ten state-of-the-art segmentation models. The assessment is multi-faceted, involving both quantitative and qualitative metrics to provide a thorough comparison. First, we delve into the performance of each model as gauged by individual metrics DSC, HD, and ASD –across the four classes of interest: Background, Brain, CSP, and LV. Next, we aggregate these metrics in a comprehensive table to offer an overall perspective on how FetSAM stands in comparison to other models. Finally, we provide a visual evaluation to supplement the quantitative metrics, showcasing the efficacy of FetSAM in real-world applications. The aim is to furnish a well-rounded view of FetSAM's performance, thereby substantiating its advantages and potential areas for improvement.

### A. Quantitative Evaluation

#### 1) Dice Similarity Coefficient (DSC)

**Fig. 3.**
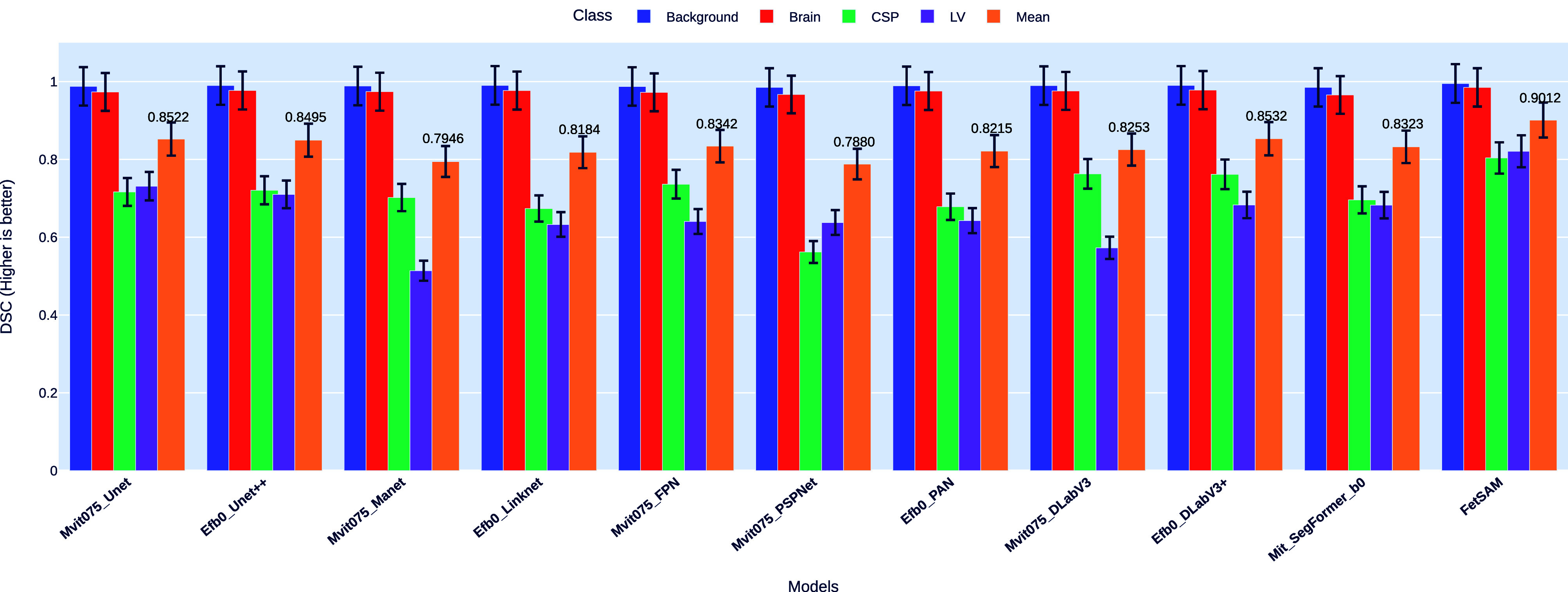
Comparison of DSC across models and classes, highlighting the superior performance of FetSAM.

DSC serves as our primary metric for evaluating segmentation accuracy, a measure that directly compares the overlap between predicted and ground truth masks. Fig. 3 visualizes these DSC scores across four classes—Background, Brain, CSP, and LV—as well as the mean DSC for each model.

##### Overall performance

Our proposed model, FetSAM, exhibits unparalleled performance, achieving a remarkable mean DSC score of 0.90117. This is substantially higher than its closest competitor, Efb0_DLabV3+, which has a mean DSC of 0.85322.

##### Class-wise insights

• *Background:* All models exhibit strong performance in segmenting the background, with DSC values above 0.98. However, FetSAM stands out with a nearly perfect DSC of 0.99506.
• *Brain:* FetSAM dominates in the Brain class as well, with a DSC of 0.98508. The nearest competing model, Efb0_DLabV3+, lags behind with a DSC of 0.97806.
• *CSP:* This class proves challenging for many models, with DSC scores as low as 0.56203 for Mvit075_PSPNet. FetSAM, however, excels with a DSC of 0.8037, substantially higher than any other model.
• *LV:* Again, FetSAM leads with a DSC of 0.82084, while the lowest performer, Mvit075_Manet, only achieves a DSC of 0.51384.

##### Comparative analysis

The consistently high DSC scores across all classes underline FetSAM's advanced segmentation capabilities. Even in more complex classes like CSP and LV, FetSAM demonstrates a significant margin of superiority over other models. The closest competitor, Efb0_DLabV3+, does well but falls short in these challenging classes.

By outperforming all other models in each individual class and on average, FetSAM validates its effectiveness and reliability for ultrasound image segmentation tasks.

#### 2) Hausdorff Distance (HD)

**Fig. 4.**
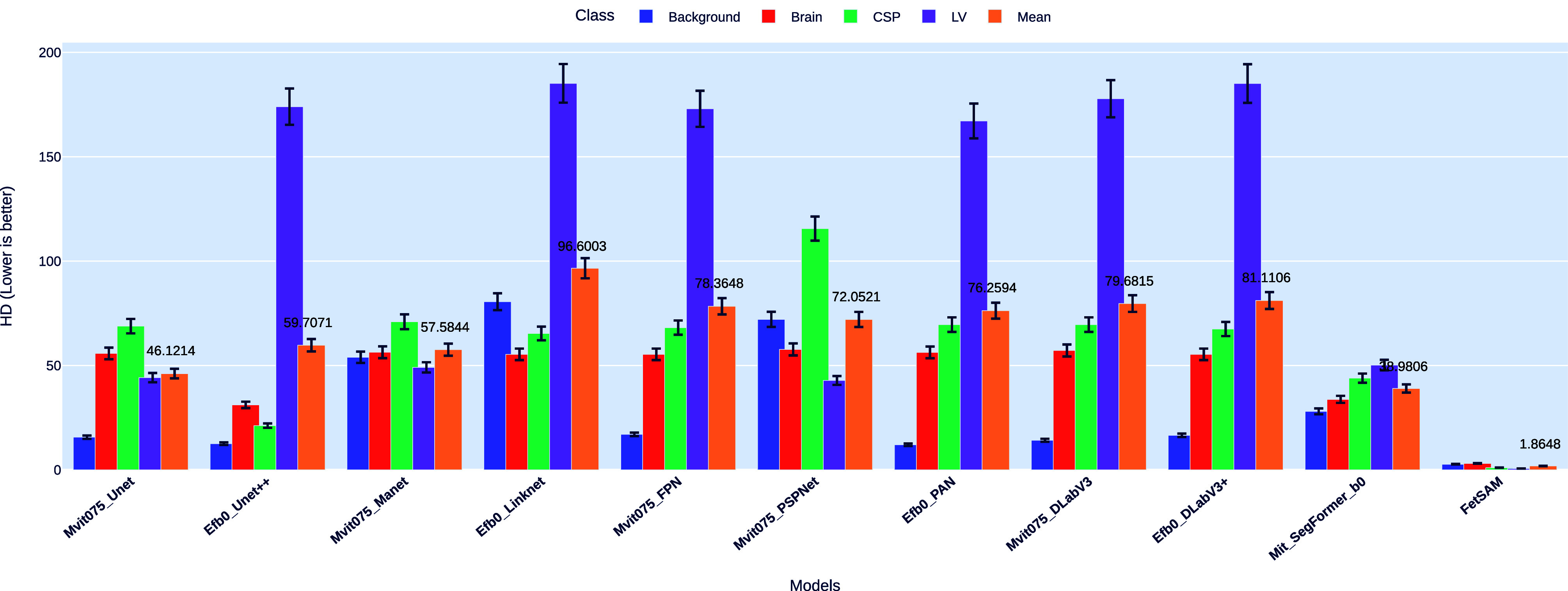
Comparison of HD across models and classes, highlighting the superior performance of FetSAM.

The Hausdorff Distance (HD) is a pivotal metric for evaluating the segmentation models. It measures the maximum distance of a set to the nearest point in the other set and focuses on the worst-case distance between the predicted segmentation and the ground truth. This attribute makes HD especially useful for understanding the model's performance when errors occur. Fig. 4 offers a detailed comparison of HD performance across various models and classes.

##### Outstanding performance of FetSAM

FetSAM excels in the HD metric, registering the lowest mean HD value of 1.86484 across all classes. This performance is notably better than the second-best model, Mit_SegFormer_b0, with a mean HD of 38.98057. FetSAM's individual class HD values are 2.67867 for Background, 3.07074 for Brain, 1.05123 for CSP, and 0.65873 for LV.

##### Role of prompts in FetSAM

The use of prompts in FetSAM appears to focus the model's attention more effectively around the Region of Interest (ROI). This focused attention is likely a significant factor contributing to FetSAM's exceptional performance in minimizing the HD.

##### Comparative analysis

On the other end of the performance spectrum, Efb0_Linknet has the highest mean HD of 96.60033, indicating less accurate segmentations in worst-case scenarios. Other models like Efb0_DLabV3+ and Mvit075_DLabV3 also demonstrated higher mean HD values, specifically 81.11064 and 79.6815, respectively.

##### Potential Areas for Improvement

Although FetSAM sets the standard in HD performance, there remains room for improvement in other models. The LV class consistently shows higher HD values, suggesting a focus area for future model enhancements.

#### 3) Average Surface Distance (ASD)

**Fig. 5.**
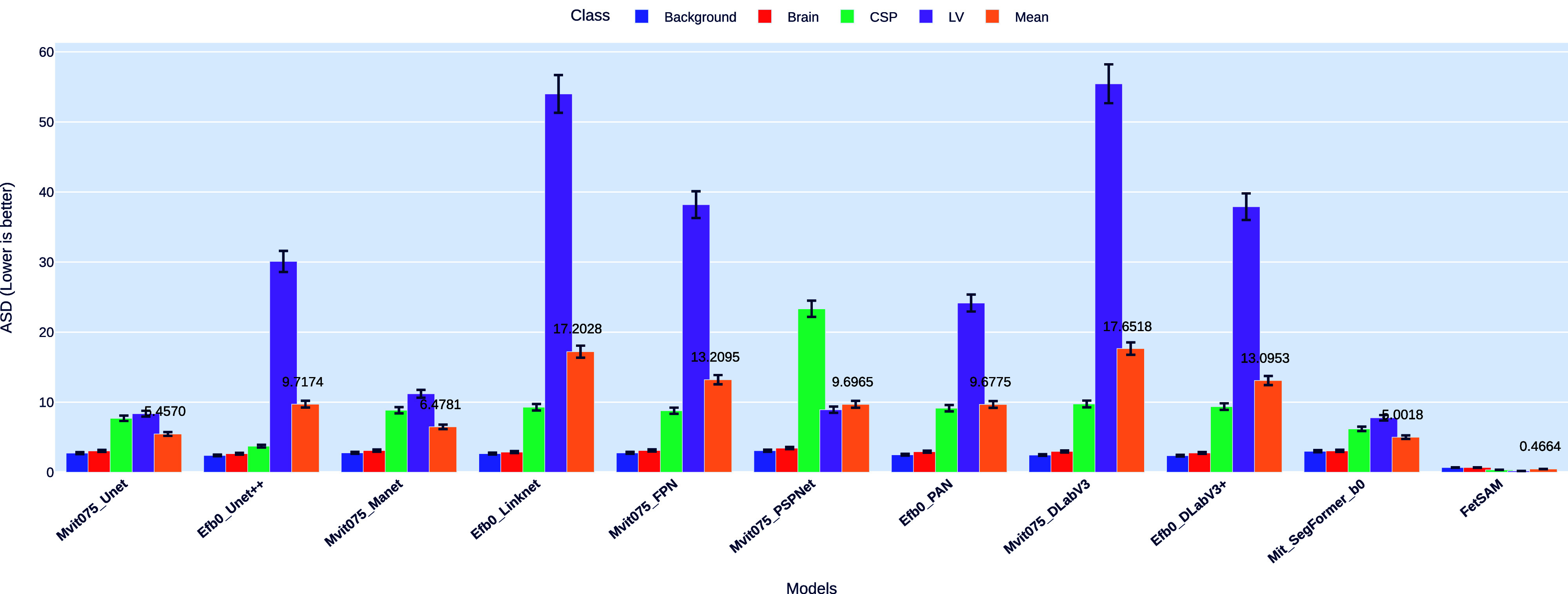
Comparison of ASD Across Models and Classes, Emphasizing the Exceptional Performance of FetSAM.

ASD is another metric of importance in evaluating the performance of segmentation models. This metric computes the mean distance between the points on the predicted segmentation and their closest points on the ground truth. Fig. 5 shows a bar chart comparison of ASD across different models and classes.

##### Exceptional performance of FetSAM

FetSAM demonstrates a spectacular performance in the ASD metric, with a mean ASD of just 0.46645. This is a considerable improvement over the next best-performing model, Mit_SegFormer_b0, which has a mean ASD of 5.0018. The individual class ASD values for FetSAM are 0.67628 for Background, 0.67105 for Brain, 0.33891 for CSP, and 0.17955 for LV.

##### Comparative analysis

Efb0_Linknet and Mvit075_DLabV3 have the highest mean ASD values, registering 17.20281 and 17.65179 respectively. These models particularly struggle in the LV class, with ASD values of 53.99729 for Efb0_Linknet and 55.44369 for Mvit075_DLabV3, pushing their mean ASD values significantly higher.

##### Potential areas for improvement

While FetSAM sets a high standard in ASD performance, other models show a wide range of ASD values, indicating room for improvement. The LV class seems to be the most problematic for many models, suggesting a potential focus area for future optimizations.

### B. Comprehensive Model Comparison

In this section, we discuss the overall performance of the ten segmentation models along with FetSAM, based on four key evaluation metrics: DSC, HD, and ASD. Table I and Table II summarizes the performance metrics for each model across the different classes: Background, Brain, CSP, and LV.

**TABLE I:** Comparison of Segmentation Performance Metrics for the Classes of Background, Brain, and CSP Across Various Models Including FetSAM

**Classes**	**Background**			**Brain**			**CSP**		
**Models**	DSC	HD	ASD	DSC	HD	ASD	DSC	HD	ASD
**Mvit075_Unet**	0.9878	15.65248	2.72715	0.97348	55.7853	3.04045	0.71627	68.85492	7.701
**Efb0_Unet++**	0.98993	12.52996	2.4004	0.97728	31.12877	2.64934	0.72059	21.18962	3.72599
**Mvit075_Manet**	0.98864	53.93515	2.76881	0.97408	56.35601	3.09529	0.70199	70.96478	8.85233
**Efb0_Linknet**	0.99025	80.56674	2.66548	0.97683	55.33534	2.8698	0.67373	65.34524	9.27866
**Mvit075_FPN**	0.98759	17	2.76385	0.97246	55.33534	3.10891	0.73634	68.1469	8.77701
**Mvit075_PSPNet**	0.98512	72.09716	3.0783	0.96697	57.69749	3.45061	0.56203	115.57681	23.33148
**Efb0_PAN**	0.98923	12.04159	2.49513	0.97575	56.26722	2.92333	0.67835	69.57011	9.14236
**Mvit075_DLabV3**	0.98961	14.17745	2.45137	0.976	57.17517	2.96622	0.76285	69.57011	9.74586
**Efb0_DLabV3+**	0.99026	16.55295	2.3647	0.97806	55.33534	2.74839	0.76165	67.47592	9.37247
**Mit_SegFormer_b0**	0.9852	28.02345	3.0031	0.96559	33.7715	3.0468	0.69608	43.94017	6.19088
**FetSAM**	**0.99506**	**2.67867**	**0.67628**	**0.98508**	**3.07074**	**0.67105**	**0.8037**	**1.05123**	**0.33891**

**TABLE II:** Comparison of Segmentation Performance Metrics for the Classes of LV and Mean Across Various Models Including FetSAM

**Classes**	**LV**			**Mean**		
**Models**	DSC	HD	ASD	DSC	HD	ASD
**Mvit075_Unet**	0.7313	44.19276	8.35949	0.85221	46.12136	5.45702
**Efb0_Unet++**	0.71013	173.97989	30.09407	0.84948	59.70706	9.71745
**Mvit075_Manet**	0.51384	49.08157	11.19606	0.79464	57.58437	6.47812
**Efb0_Linknet**	0.63277	185.15399	53.99729	0.81839	96.60033	17.20281
**Mvit075_FPN**	0.64038	172.97688	38.18823	0.83419	78.36478	13.2095
**Mvit075_PSPNet**	0.63774	42.8369	8.92578	0.78796	72.05209	9.69654
**Efb0_PAN**	0.64272	167.1586	24.14913	0.82151	76.25938	9.67749
**Mvit075_DLabV3**	0.57274	177.80327	55.44369	0.8253	79.6815	17.65179
**Efb0_DLabV3+**	0.68293	185.07837	37.89552	0.85322	81.11064	13.09527
**Mit_SegFormer_b0**	0.68247	50.18713	7.76644	0.83234	38.98057	5.0018
**FetSAM**	**0.82084**	**0.65873**	**0.17955**	**0.90117**	**1.86484**	**0.46645**

FetSAM emerges as the standout model, leading in three out of the four metrics. It achieves a mean DSC value of 0.90117, an HD value of 1.86484, and an ASD value of 0.46645.

Among the other models, Efb0_DLabV3+ performs well in terms of DSC struggles in HD and ASD. On the contrary, models like Mvit075_DLabV3 and Efb0_Linknet show generally poor performance across most metrics. Their lesser performance is most evident in the LV class, thereby affecting their overall mean metric values.

A consistent trend observed across most models is their similar performance in DSC and ASD, but a noticeable struggle in HD. This suggests that while these models are generally good at segmentation, they are prone to larger errors. This makes FetSAM's robustness even more commendable as it maintains low error rates across all these metrics.

It's worth noting that the LV class is challenging for almost all models, particularly in terms of HD. This could be an area for future research and refinement of segmentation models.

The superior performance of FetSAM is likely due to its use of prompts, which allow the model to focus more on the ROI. This focused attention mechanism is a likely contributor to its overall exceptional performance across metrics.

In conclusion, while FetSAM sets a high standard in segmentation performance, there is room for improvement in other models, especially in the HD and ASD metrics and in challenging classes like LV.

### C. Ablation Studies on Loss Functions and Model Configuration

To assess the effectiveness of the chosen loss functions and the robustness of the FetSAM model under various configurations, extensive ablation studies were undertaken. These studies not only evaluate the individual contributions of the Dice and Lovasz loss functions but also investigate their combined effect. Additionally, the adaptability and consistent performance of FetSAM with different input prompts are analyzed. The findings from these ablation studies are essential to validate the design decisions made in developing FetSAM and to demonstrate its enhanced performance in fetal ultrasound image segmentation.

#### 1) Impact of Loss Function on Segmentation Performance

The ablation study, presented in Table III, aimed to assess the impact of various loss functions on the performance of different segmentation models. The Dice Loss serves as a baseline, while the Lovasz Loss, targeting the optimization of the intersection-over-union metric, offers a nuanced approach. Our findings suggest that a Combined Loss function, integrating both Dice and Lovasz Losses, significantly outperforms the individual loss functions across all models. This combination notably improves the DSC and reduces the HD and ASD, underscoring its effectiveness in enhancing segmentation precision.

**TABLE III:** Ablation Study Results for Different Loss Functions

**Model**	**Loss Function**	**DSC**	**HD**	**ASD**
Mvit075 Unet	Dice Loss	0.73273	98.89012	24.47232
Mvit075 Unet	Lovasz Loss	0.82148	90.42007	15.122
Mvit075 Unet	Combined Loss	0.85221	46.12136	5.45702
Efb0 Unet++	Dice Loss	0.79074	92.83841	15.01829
Efb0 Unet++	Lovasz Loss	0.75743	80.56693	18.68003
Efb0 Unet++	Combined Loss	0.84948	59.70706	9.71745
Mvit075 Manet	Dice Loss	0.8166	106.6184	17.99852
Mvit075 Manet	Lovasz Loss	0.82439	91.28148	15.51446
Mvit075 Manet	Combined Loss	0.79464	57.58437	6.47812
Efb0 Linknet	Dice Loss	0.80538	97.69617	18.1788
Efb0 Linknet	Lovasz Loss	0.78093	98.77628	19.81796
Efb0 Linknet	Combined Loss	0.81839	96.60033	17.20281
Mvit075 FPN	Dice Loss	0.77973	103.75494	19.41409
Mvit075 FPN	Lovasz Loss	0.7659	80.353	15.23116
Mvit075 FPN	Combined Loss	0.83419	78.36478	13.2095
Mvit075 PSPNet	Dice Loss	0.79621	102.00329	10.53926
Mvit075 PSPNet	Lovasz Loss	0.77763	92.76895	19.1604
Mvit075 PSPNet	Combined Loss	0.78796	72.05209	9.69654
Efb0 PAN	Dice Loss	0.78456	78.18214	15.83406
Efb0 PAN	Lovasz Loss	0.77892	98.14483	18.13836
Efb0 PAN	Combined Loss	0.82151	76.25938	9.67749
Mvit075 DLabV3	Dice Loss	0.79225	99.4184	18.57266
Mvit075 DLabV3	Lovasz Loss	0.81267	80.47812	19.10652
Mvit075 DLabV3	Combined Loss	0.8253	79.6815	17.65179
Efb0 DLabV3+	Dice Loss	0.82243	82.76645	15.54855
Efb0 DLabV3+	Lovasz Loss	0.7792	83.84735	17.03116
Efb0 DLabV3+	Combined Loss	0.85322	81.11064	13.09527
Mit SegFormer b0	Dice Loss	0.8294	39.82529	5.41315
Mit SegFormer b0	Lovasz Loss	0.82167	42.48376	5.2876
Mit SegFormer b0	Combined Loss	0.83234	38.98057	5.0018
FetSAM	Dice Loss	0.86675	5.09663	1.49432
FetSAM	Lovasz Loss	0.883960	3.480735	0.980385
FetSAM	Combined Loss	**0.90117**	**1.86484**	**0.46645**

The Study Compares the Performance of Various Segmentation Models Using Dice Loss, Lovasz Loss, and a Combined Loss of Dice and Lovasz. Performance is Evaluated Based on DSC, HD, and ASD.

#### 2) Ablation Study on FetSAM's Sensitivity to Prompt Size

In our comprehensive ablation study, we scrutinized the influence of prompt size on FetSAM's segmentation proficiency. This examination is pivotal for fetal biometrics in prenatal diagnostics, where precision is paramount. We initially deployed FetSAM with bounding boxes formulated by our algorithm, then we probed its adaptability to varying prompt dimensions by expanding the bounding box offsets to 0, 10, and 20, respectively.

Table IV delineates a discernible performance degradation concomitant with increased offsets. Operating with zero offset, FetSAM showcases exemplary efficacy across the board. Yet, as offsets extend to 10 and 20, a conspicuous downturn is observed in DSC along with escalations in both HD and ASD metrics, a trend that is especially pronounced within the LV class – crucial for the fidelity of biometric computations. These results articulate the criticality of prompt exactitude for FetSAM's optimal utilization in clinical milieus, which demands not merely segmentation, but also the scrupulous reckoning of biometrics. Armed with precise bounding prompts, FetSAM transcends conventional models, bestowing enhanced HD and ASD results. Such findings illuminate the exigencies of optimizing FetSAM for variable prompt dimensions, bolstering its versatility and clinical viability. It is this capability for precise biometric elucidation that positions FetSAM as a harbinger for transformation in fetal medicine, equipping practitioners with data of unparalleled reliability for the assessment of fetal well-being and progression.

**TABLE IV:** Ablation Study Results for FetSAM Model With Baseline and Varying Prompt Sizes

Model	Mode	Prompt's Offset	Background			Brain			CSP			LV			Mean		
			DSC	HD	ASD	DSC	HD	ASD	DSC	HD	ASD	DSC	HD	ASD	DSC	HD	ASD
FetSAM	Baseline	0	0.89458	90.57674	18.99524	0.70289	51.5836	15.66214	0.26425	13.45299	9.28836	0.53828	37.31849	31.65951	0.6	45.73295	16.90131
FetSAM	Fine-tuned	0	**0.99506**	**2.67867**	**0.67628**	**0.98508**	**3.07074**	**0.67105**	**0.8037**	**1.05123**	**0.33891**	**0.82084**	**0.65873**	**0.17955**	**0.90117**	**1.86484**	**0.46645**
FetSAM	Fine-tuned	10	0.93892	12.35367	8.53346	0.87781	12.38732	8.17055	0.63352	4.13365	2.53643	0.74456	2.30408	1.28159	0.7987	7.79468	5.13051
FetSAM	Fine-tuned	20	0.89018	22.83587	14.71003	0.78481	23.1562	14.58581	0.51974	8.6076	6.14355	0.68114	4.75064	3.35741	0.71897	14.83758	9.6992

Adding to our insights, the inclusion of baseline results for FetSAM before fine-tuning, as depicted in the augmented Table 4, further substantiates the significant enhancements post-tuning. These enhancements are not trivial; they accentuate the transformative impact of fine-tuning in advancing FetSAM from a baseline model to one that sets new precedents in fetal biometric segmentation. As we march towards clinical application, these ablation studies serve as a testament to FetSAM's potential in real-world settings, where precision is not a luxury but a necessity.

### D. Qualitative Evaluation and Visual Comparison

**Fig. 6.**
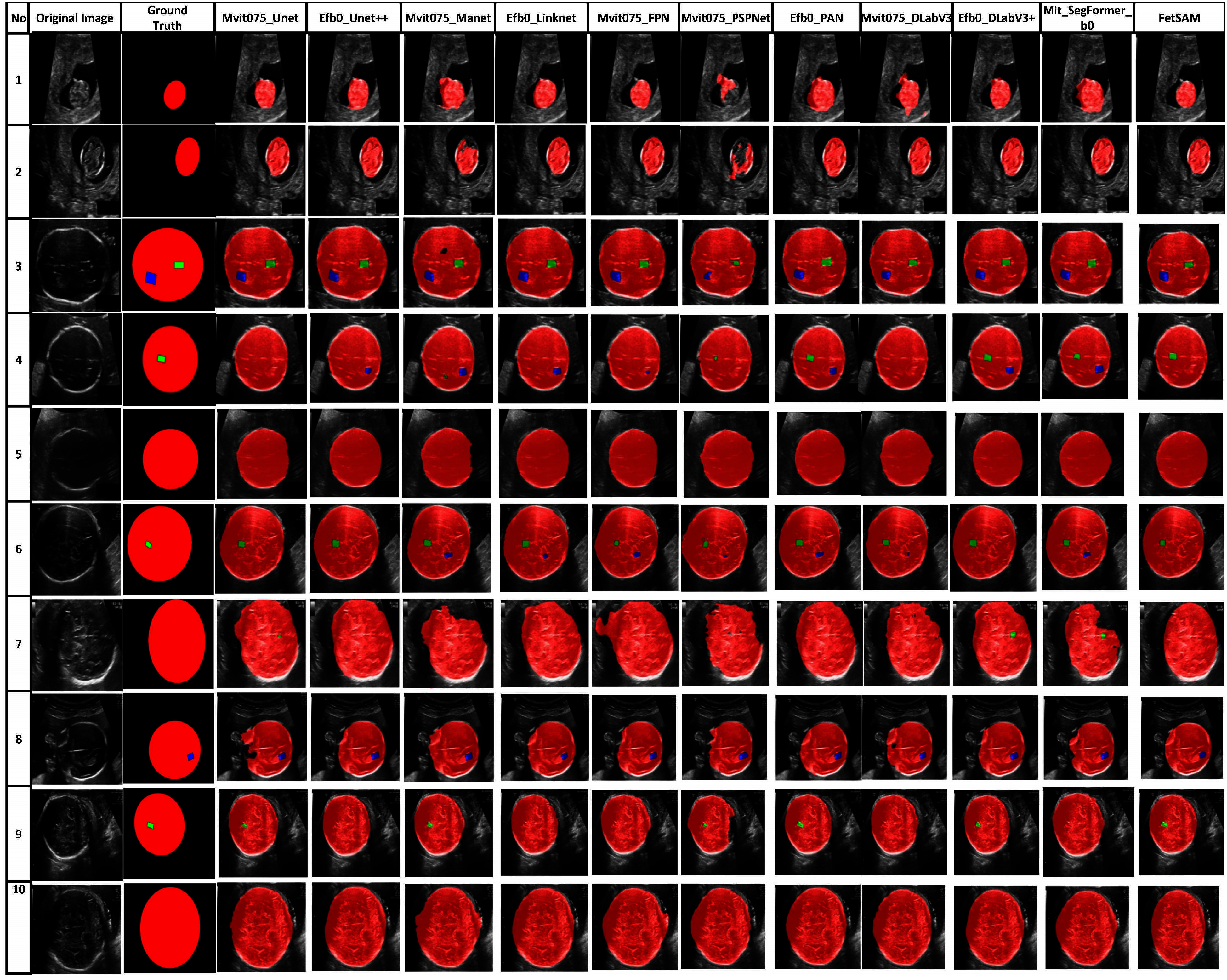
Comparison of predicted segmentation masks: A side-by-side comparison of the 10 masks produced by each segmentation model, contrasted with the ground truth and our proposed FetSAM model.

Fig. 6 provides a side-by-side comparison of the segmentation masks produced by each model, including our proposed FetSAM model. This qualitative comparison is not just an accessory but a critical aspect of our evaluation, as it allows us to understand the nuanced performance of each model in various scenarios that occur in different trimesters of fetal development. In the first and second images, which focus on the fetal brain during the first trimester, FetSAM stands out for its exceptional performance. This is noteworthy because CSP and LV are not yet visible at this stage. Other models that follow in terms of accuracy are Efb0_DLabV3+, Mit_SegFormer_b0, Mvit075_Unet, Mvit075_FPN, and Efb0_Linknet.

Moving on to images 3, 4, and 5, which capture the fetal head in the second trimester, FetSAM continues to impress. Not only does it accurately predict the fetal brain, but it also does an excellent job with the CSP and LV, which have now become visible. Models like Mit_SegFormer_b0, Mvit075_Unet, Mvit075_DLabV3, and Mvit075_FPN also perform well but lag behind FetSAM. For instance, image 4 reveals that while some models, such as Efb0_Unet++ and Mvit075_Manet, incorrectly predict LV, FetSAM remains consistent with the ground truth. This consistency is also observed in models like Efb0_PAN, Efb0_DLabV3+, and Mit_SegFormer_b0.

The complexity increases with images 7 and 8, representing the third and second trimesters, respectively. Here, many models struggle to predict the complete shape of the fetal brain. Yet, FetSAM, along with Mvit075_Unet and Efb0_PAN, rises to the challenge in image 7. In image 8, FetSAM alone predicts the correct mask accurately, followed by Efb0_PAN and Efb0_Linknet.

Lastly, image 9 brings a unique challenge due to the different orientation of the fetal brain, which complicates CSP detection. Nevertheless, FetSAM, along with Efb0_DLabV3+, Efb0_PAN, Mvit075_Unet, and Mvit075_Manet, manages to handle this complexity effectively.

The visual observations confirm our quantitative findings, further emphasizing FetSAM's robustness and adaptability across different developmental stages and imaging conditions.

## IV. Discussion

In this work, we have embarked on a pioneering study in the domain of fetal brain segmentation, introducing the innovative FetSAM model. Utilizing a novel dataset crafted specifically for this study, our research stands as a groundbreaking effort in the field, marked by the absence of pre-existing benchmarks in the literature. This uniqueness renders our findings exceptionally impactful.

The FetSAM model has demonstrated superior performance across key quantitative metrics such as the Dice Similarity Coefficient (DSC), Hausdorff Distance (HD), and Average Surface Distance (ASD). This high level of performance is attributed to its prompt-based architecture, which facilitates targeted attention on regions of interest (ROI). Such precision is crucial in complex anatomical tasks like fetal brain segmentation, where anatomical structures undergo significant changes across different trimesters.

Our methodological approach was robust, incorporating class weighting, custom loss functions, and extensive data augmentation to address the challenges posed by the imbalanced dataset, particularly for the LV class. Despite these enhancements, areas for improvement remain, particularly in the DSC and HD metrics, indicating that the LV class could be a focal point for future research and optimization.

While models such as Efb0_DLabV3+ and Mvit075_ DLabV3 exhibited competitive performance in certain metrics, they were consistently outperformed by FetSAM, especially in more challenging scenarios like third-trimester images or varying fetal brain orientations. These observations underscore FetSAM's robustness and its potential for both further research and practical clinical applications.

Notably, all models faced challenges in accurately segmenting the LV class, highlighting a need for future research to focus on achieving a more balanced dataset and possibly developing more tailored attention mechanisms or custom loss functions.

The ablation studies conducted have further validated FetSAM's advanced segmentation capabilities, particularly its utilization of prompt boxes to enhance segmentation precision. This novel approach significantly reduces false positives and false negatives, contributing to the model's exceptional performance in the HD metric. Such improvements are critical for clinical applications where precise segmentation of edges and small structures directly influences diagnostic outcomes.

The remarkable HD improvement, outpacing the enhancements seen in DSC, highlights the method's efficacy in precise edge and structure delineation. While DSC measures overall accuracy, HD's sensitivity to object boundary delineation makes FetSAM's focus on minimizing segmentation errors in these areas a key factor in its superior HD performance.

Ultimately, FetSAM's innovative prompt box utilization not only enhances segmentation accuracy but also illustrates its potential to significantly advance medical image segmentation. Future efforts will aim to further refine these techniques, improving FetSAM's robustness and clinical utility across diverse scenarios.

In conclusion, FetSAM sets a new benchmark in fetal brain segmentation, offering a solid foundation for future research in this field. Future studies might explore alternative attention mechanisms, advanced data augmentation techniques, and specialized loss functions to further refine FetSAM's performance, particularly in areas identified as needing improvement.

## V. Conclusion

This study marks a significant advancement in fetal brain segmentation by unveiling the FetSAM model, which is calibrated on a novel dataset and juxtaposed with a multitude of segmentation models. FetSAM has demonstrated its robustness and adaptability, excelling in critical quantitative metrics and showcasing potential for real-world applications due to its superior qualitative performance.

Looking ahead, we recognize the importance of transitioning towards fully automated systems that encapsulate the entire process from frame selection to segmentation, catering to the emerging trends in the field. The current success of FetSAM illustrates its potential to adapt to such an end-to-end automated framework, which would accommodate variations in sonographer expertise–from novices to seasoned practitioners–and enhance the consistency of fetal brain segmentation outcomes.

To further enhance FetSAM, our immediate research goals include experimenting with diverse types of prompts for the model, ranging from textual to point-based inputs, to optimize segmentation accuracy. Moreover, a comparative analysis between FetSAM's performance and that of fetal ultrasound sonographers is envisaged. Such a study would deepen our understanding of FetSAM's utility in clinical scenarios and benchmark its efficacy against the human expertise that currently defines the standard of care.

In sum, the FetSAM model establishes a solid groundwork for both immediate usage and future enhancements in the arena of fetal brain segmentation. Its commendable performance, allied with the scope for further improvements, positions it as a strong prospect for exhaustive clinical trials and applications in real-world healthcare settings.

## Acknowledgment

This research did not receive any specific grant from funding agencies in the public, commercial, or not-for-profit sectors. However, Open Access funding was provided by the Qatar National Library.

## Availability of Data and Materials

The datasets generated and analysed during the current study are available in the Zenodo repository, https://doi.org/10.5281/zenodo.8265464. The corresponding author can be contacted for any further access if required.

## Conflict of Interest

The authors affirm that this study was carried out without any commercial or financial associations that would be interpreted as indicative of potential conflicts of interest.

## Author Contributions

Data curation was handled by MAL. Formal analysis was conducted by MAL and US. MAL and US led the investigation and developed the methodology. MH administered the project, while MAL provided the resources. Supervision was carried out by MA and MH. MAL was responsible for validation and visualization. The original draft was written by MAL, and the manuscript was reviewed and edited by US,MA, and MH. All authors read and approved the final manuscript.
